# Motion-Driven Automatic IMU Orientation Calibration via *SO*(3) Pattern Alignment

**DOI:** 10.3390/s26144342

**Published:** 2026-07-08

**Authors:** Anik Sarker, Alan T. Asbeck

**Affiliations:** Department of Mechanical Engineering, Virginia Tech, Blacksburg, VA 24060, USA; aniks@vt.edu

**Keywords:** sensor-to-segment calibration, inertial measurement unit, IMU calibration, rotation pattern alignment, coordinate frame alignment, orientation distribution

## Abstract

**Highlights:**

**What are the main findings?**

- We propose and validate a novel method to automatically calibrate IMUs without a dedicated calibration step.
- Walking, typing, and using a mouse are the activities of daily living that lead to the best automatic calibration for IMUs on the pelvis and wrist.

**What are the implications of the main findings?**

- If an activity (e.g., walking) is identified, a worn sensor’s orientation can be automatically calibrated with accuracy comparable to using a dedicated calibration procedure.

**Abstract:**

Calibration of body-worn inertial measurement units (IMUs) is essential for accurate motion estimation, yet sensor orientations may drift over time due to slippage or long-term integration errors. This causes an IMU to gradually lose alignment with the body segment on which it is mounted. In this paper, we develop a novel technique for Automatic Calibration—recovering this alignment during normal motion, without a dedicated calibration step. We implement automatic calibration by formulating it as the alignment of motion-induced orientation distributions on SO(3). When the activity being performed is known (e.g., walking), the distribution of orientations for a correctly calibrated IMU exhibits a characteristic and repeatable pattern on SO(3). Although sensor drift may rotate this distribution into a different orientation, the underlying pattern remains similar. Thus, we calibrate a drifted IMU by aligning its observed orientation distribution to a prior reference distribution obtained from the same activity, either from the same subject at an earlier time or from other subjects. To perform this alignment, we employ a correspondence-free spherical pattern matching method on SO(3) (SO3_SPMC), based on transformed basis vector distributions and spherical cross-correlation. We evaluate the proposed automatic calibration framework with N = 5 people wearing body-worn IMUs during activities of daily living, demonstrating calibration accuracy comparable to supervised single-frame calibration without requiring explicit calibration poses or user intervention. The activities of walking, typing, and using using a computer mouse were best for automatically calibrating a sensor on the wrist.

## 1. Introduction

Aligning the coordinate frame of a sensor or object with a known reference frame through a rotation is a fundamental problem in robotics and computer vision. Given vector observations expressed in different coordinate frames, the objective is to estimate the rotation that best aligns these observations across frames. This problem arises in a wide range of applications, including point-cloud registration [1,2,3]; multi-modal calibration [4,5,6,7,8], state estimation [9,10,11,12], and more.

A particularly important instance of this problem is the calibration of Inertial Measurement Units (IMUs) with respect to a known reference frame, such as a human body segment or a robot link. This extrinsic calibration from an IMU to a body segment is often called sensor-to-segment calibration. In typical calibration procedures, the IMU’s coordinate frame is initially unknown and is determined through a dedicated calibration step, often requiring the user or robot to assume one or more specific poses. For body-worn sensors, this commonly involves having the wearer stand in predefined postures while data are collected and used to estimate a fixed rotation between the sensor frame and the body segment frame. Similar procedures are used for robot-mounted or standalone IMUs by placing the sensor in known orientations. Such approaches have been widely studied [13,14]. In this paper, we refer to calibration procedures that rely on a single pose or time instant as *Single Frame Calibration*.

Several alternative strategies have also been proposed. For example, ref. [15] estimated the orientation of a worn IMU while a person was riding in a car, a combination of visual sensors and IMUs was used by [16,17,18,19], and a model-based approach was used by [20,21]. Other approaches exploit stationary periods to perform recalibration, such as Zero Velocity Update (ZUPT) methods for foot-mounted IMUs [22] or wrist-worn IMUs [23]. In contrast, some recent works bypass explicit calibration altogether by directly learning task-specific mappings from raw IMU signals using machine learning techniques [24,25,26]. While effective for specific applications, such approaches often sacrifice physical interpretability and generalizability.

In this work, we focus on IMUs worn on the human body for the purpose of estimating body kinematics, although our approach is applicable to any sensor that undergoes largely repeatable motion patterns. Accurate interpretation of such data requires knowledge of each sensor’s orientation relative to the body segment (or other object) on which it is mounted. While this orientation can be determined through an explicit calibration step, doing so is often inconvenient in practice. Moreover, even when an initial calibration is performed, the sensor may lose calibration over time due to slippage, impacts, or long-term drift during physical activity. This can occur during sports [27,28,29,30,31], or during all-day wear when monitoring human motion in industrial [32] or health [33,34,35] contexts. Automatically correcting this loss of calibration during motion, without requiring any specific user actions, would therefore be highly desirable. We refer to this problem as *Automatic Calibration*.

The key observation underlying our approach is that Automatic Calibration can be formulated as an orientation distribution alignment problem. During a known activity (e.g., walking), a correctly calibrated body-worn IMU produces a characteristic and repeatable distribution of orientations on SO(3).  Although sensor drift may rotate this distribution into a different orientation, the overall pattern induced by the activity remains similar. By exploiting this structure, a drifted IMU can be recalibrated by aligning its observed orientation distribution to a prior reference distribution obtained from the same activity, either from the same person at an earlier time or from other people performing the same activity. This formulation requires aligning distributions on SO(3) rather than relying on frame-to-frame correspondences.

To demonstrate the practical relevance of this formulation, we consider a real-world motion capture setting involving body-worn IMUs. State-of-the-art whole-body IMU-based motion capture systems, such as the XSens MVN Link system (“MVN”, Movella, Inc., Henderson, NV, USA), achieve high accuracy, with reported absolute mean orientation errors below 5° when benchmarked against optical motion capture [36,37,38,39,40]. However, such systems are expensive (approximately USD 12,000  per unit) and cumbersome to deploy, limiting their usability in many practical scenarios such as rehabilitation studies.

As a cost-effective alternative, standalone IMUs such as the XSens DOT sensor (“DOT”) [41] can be used in conjunction with motion inference algorithms [42,43]. However, unlike integrated systems such as MVN, standalone sensors exhibit significant variability in their orientation outputs across sessions, even when placed at the same physical location and orientation. In practice, this variability can result in orientation errors as large as 90°. Our objective is therefore to automatically calibrate the orientation of standalone sensors to the MVN reference frame without requiring explicit calibration poses. This is particularly valuable given the prevalence of datasets [44] and learning-based models that rely on MVN or similar systems [42,45,46]. Mapping standalone sensor data into the MVN reference frame enables direct reuse of such datasets and models.

The contributions of this paper are as follows: We formulate Automatic Calibration of body-worn IMUs as the alignment of motion-induced orientation distributions on SO(3), exploiting the repeatability of activity-specific orientation patterns. We present a practical Automatic Calibration pipeline that aligns drifted orientation distributions from standalone IMUs to prior reference distributions without assuming frame-to-frame correspondences. This pipeline uses an existing SO(3) distribution alignment method to automatically calibrate IMUs during normal daily motion. We validate our proposed approach by conducting real-world experiments with body-worn IMUs during activities of daily living. We demonstrate that our new method leads to calibration accuracy comparable to that of supervised Single Frame Calibration, but without requiring explicit calibration procedures.

## 2. Materials and Methods

### 2.1. Concepts and Notations

#### 2.1.1. Rotating 3D Cartesian Points

Let the points in $P\in R^{n\times 3}$ and let R∈SO(3). Applying the same rotation to all points uses right-multiplication:

(1) $$P_{\mathrm{rotated}}\;=\;P\;R.$$

#### 2.1.2. Rows vs. Columns of a Rotation Matrix

For the active, column-vector convention $v^{\prime }=R\;v$ with R∈SO(3):

- Columns: column *j* equals $R\;{\hat{e}}_{j}$; i.e., the rotated canonical axes expressed in the original frame.
- Rows: row *i* equals ${\hat{e}}_{i}^{⊤}R$, giving components via ${\left(v^{\prime }\right)}_{i}={\mathrm{row}}_{i}\left(R\right)\cdot v$; geometrically, original-frame axes expressed in the rotated frame (since $R^{-1}=R^{⊤}$).

Example: a *z*-axis rotation by θ,

$$R_{z}\left(\theta \right)=\left[\begin{array}{ccc} \cos\theta & -\sin\theta & 0\\ \sin\theta & \;\cos\theta & 0\\ 0 & 0 & 1 \end{array}\right],$$

has first/second columns as the rotated *x*/*y* axes, and the first row [cosθ,−sinθ,0] extracts the *x*-component after rotation.

#### 2.1.3. Transformed Basis Vectors (TBVs)

Let $R_{A\leftarrow B}\in SO\left(3\right)$ map coordinates from frame *B* to frame *A*, i.e., $v_{A}=R_{A\leftarrow B}v_{B}$. We define *Transformed Basis Vectors (TBVs)* using the rows of $R_{A\leftarrow B}$:

$${\mathrm{TBV}}_{i}\;≜\;{\mathrm{row}}_{i}\;{\left(R_{A\leftarrow B}\right)}^{⊤}={\left(R_{A\leftarrow B}\right)}^{⊤}{\hat{e}}_{i}^{A},\;i\in \left\{x,y,z\right\}.$$

Thus, ${\mathrm{TBV}}_{x}$, ${\mathrm{TBV}}_{y}$, and ${\mathrm{TBV}}_{z}$ are the first, second, and third rows of R (transposed), respectively; equivalently, they are the columns of $R^{⊤}$. Geometrically, ${\mathrm{TBV}}_{i}$ is frame *A*’s *i*-axis expressed in frame *B*. Because R∈SO(3), the TBVs form an orthonormal triad. For clarity, the *columns* of $R_{A\leftarrow B}$ are frame *B*’s axes expressed in frame *A*, and the entries satisfy ${\left(R_{A\leftarrow B}\right)}_{ij}={\hat{e}}_{i}^{A}\;\cdot \;{\hat{e}}_{j}^{B}$. In computation, the row view provides direct component extraction,

$${\left(v_{A}\right)}_{i}\;=\;{\mathrm{row}}_{i}\;\left(R_{A\leftarrow B}\right)\;v_{B},$$

and we adopt this row-centric convention throughout unless stated otherwise.

#### 2.1.4. Set of TBVs from Set of Orientations (SO(3))

Given a set of *n* orientation samples in SO(3), we store them as an array of rotation matrices of shape n×3×3. From this array, we extract the TBVs, yielding three TBV sets: *x*-TBVs, *y*-TBVs, and *z*-TBVs.

#### 2.1.5. $S^{2}$ Representation and S2-Point: Normalized 3D Vector Representation on Sphere Surface

The *n*-sphere framework $\left(S^{n}\right)$, a family of compact manifolds embedded in $R^{n+1}$, is widely used in computer vision, including viewpoint and surface-normal estimation [47,48]. On the 2-sphere $\left(S^{2}\right)$, a unit 3D vector is represented by its endpoint on the sphere surface, which we refer to as an “S2-point” [49]. Thus, a normal vector $\vec{OP}$ of unit length corresponds to the point $P\in S^{2}$; the coordinates of *P* encode the direction of the original unit vector (see Figure 1a). The axes of a global reference frame intersect the sphere at three points (on *x*, *y*, *z*), which can likewise be viewed as the $S^{2}$ representation of that frame.

#### 2.1.6. $S^{2}$ Representation of TBVs

Typically, in the *n*-sphere paradigm, a quaternion (in $R^{4}$) is represented on $S^{3}$ [49]. Here, we instead use the $S^{2}$ representation of normal vectors for orientation-derived features. A single rotation matrix induces three S2-points via its *x*-, *y*-, and *z*-TBVs. In Figure 1a, the identity rotation yields three S2-points at the coordinate axes. For *n*SO(3) samples, we first extract three TBV sets (Section 2.1.4) and then represent them as three S2-distributions on the sphere surface, denoted $S2_{x}$, $S2_{y}$, and $S2_{z}$; see Figure 1b.

#### 2.1.7. $S^{2}$ Point-Pattern Registration

Spherical point-pattern registration seeks the optimal rotation that aligns two $S^{2}$ distributions (target and source). In our prior work [50,51], we introduced three algorithms for robust alignment of spherical signals: *SPMC* (Spherical Pattern Matching by Correlation), *FRS* (Fast Rotation Search), and a hybrid *SPMC_FRS*. These methods are correspondence-free and robust to moderate noise as well as extremely high outlier ratios. Unlike traditional solutions to the Wahba problem [52,53,54,55,56,57,58], algorithms such as *SPMC* do not require pairwise correspondences between spherical signals. Moreover, the source may contain up to 90% outliers provided the underlying signal remains sufficiently complete relative to the target. The output is a rotation that aligns the source spherical distribution to the target, which can be applied to 3D points via Equation (1).

#### 2.1.8. Axis-Consistent vs. Axis-Ambiguous

Each R∈SO(3) yields ${\mathrm{TBV}}_{x}\left(R\right)$, ${\mathrm{TBV}}_{y}\left(R\right)$, ${\mathrm{TBV}}_{z}\left(R\right)\in S^{2}$. A TBV *sign flip* is the antipodal map ${\mathrm{TBV}}_{i}\mapsto -{\mathrm{TBV}}_{i}$ (axis preserved, direction reversed). In *axis-consistent* settings (e.g., homogeneous sensors sharing a base frame), TBVs correspond one-to-one—x↔x, y↔y, z↔z—and differ only by a rotation (e.g., two XSens IMUs on the same rigid body [43]). In *axis-ambiguous* settings (e.g., robot world/hand–eye or cross-vendor rigs), axes may be *relabelled* and/or *sign-flipped* (e.g., source *x*-TBV matches target −y-TBV).

### 2.2. Problem Statement

Let $A={\left\{a_{i}\right\}}_{i=1}^{N}$ and $B={\left\{b_{j}\right\}}_{j=1}^{M}$ be two sets of 3D orientations, where $a_{i},b_{j}\in SO\left(3\right)$ and the sets are *axis-consistent*. We assume that the orientations in B are related to those in A by an unknown global rotation, such that

(2) $$b_{j}=R\;a_{j}+\epsilon_{j},$$

where R∈SO(3) is the unknown rotation to be estimated, and $\epsilon_{j}\in SO\left(3\right)$ models measurement noise, outliers, or orientation drift.

Our objective is to estimate the rotation R that best aligns the orientation distribution of B to that of A by minimizing the error across the set. Equivalently, we seek the rotation R such that a subset of samples from A explains the observed orientations in B under Equation (2), with minimal residual error.

#### Overview of the Solution

To align two axis-consistent orientation distributions, we employ the SO3_SPMC algorithm [51]. The goal of SO3_SPMC is to probabilistically align a source orientation set, denoted B, to a reference orientation set A. In the context of automatic calibration, A represents a known, correctly calibrated orientation distribution, while B corresponds to a potentially drifted distribution from the same activity.

The alignment is performed by correlating the transformed $S^{2}$ representations of the Transformed Basis Vectors (TBVs) extracted from the two orientation sets. This correspondence-free formulation allows robust estimation (rotational errors of 0.1–0.7°) even in the presence of noise, drift, and outliers. Full implementation details of the SO3_SPMC algorithm are provided in [51]; for completeness, we summarize it in Algorithm 1.
**Algorithm 1** SO3_SPMC**Inputs.** Target rotations ${\left\{R_{k}^{A}\right\}}_{k=1}^{n}$, Source rotations ${\left\{R_{\ell }^{B}\right\}}_{\ell =1}^{m}$. A per-axis spherical matcher MATCH(·,·) instantiated as *SPMC* [50].**Outputs.** Estimated global alignment $R_{\mathrm{est}}\in SO\left(3\right)$ (maps Source to Target under the row-vector convention).**Procedure.** 1. *Extract TBVs per set.*$\left(A_{x},A_{y},A_{z}\right)\;\leftarrow \;\mathrm{TBVsfromSO}3\left(\left\{R_{k}^{A}\right\}\right)$,$\left(B_{x},B_{y},B_{z}\right)\;\leftarrow \;\mathrm{TBVsfromSO}3\left(\left\{R_{\ell }^{B}\right\}\right)$.Each $A_{i},B_{i}$ is an $S^{2}$ point set (row-centric TBVs; Section 2.1.3). 2. *Align each axis via the chosen matcher.*$\left(\text{\_},{\tilde{B}}_{x}\right)\;\leftarrow \;\mathrm{MATCH}\left(A_{x},B_{x}\right)$, $\left(\text{\_},{\tilde{B}}_{y}\right)\;\leftarrow \;\mathrm{MATCH}\left(A_{y},B_{y}\right)$, $\left(\text{\_},{\tilde{B}}_{z}\right)\;\leftarrow \;\mathrm{MATCH}\left(A_{z},B_{z}\right)$. 3. *Form mean frames.*$\mathrm{MeanAligned}=\left[\mu \left({\tilde{B}}_{x}\right);\mu \left({\tilde{B}}_{y}\right);\mu \left({\tilde{B}}_{z}\right)\right]$,$\mathrm{MeanSource}=\left[\mu \left(B_{x}\right);\mu \left(B_{y}\right);\mu \left(B_{z}\right)\right]$,where μ(·) is the Euclidean mean (normalize if desired). 4. *Estimate global rotation (Procrustes projection).*$R_{\mathrm{frame}}=\Pi_{SO(3)}\;\left({\mathrm{MeanAligned}}^{⊤}\mathrm{MeanSource}\right)$,$R_{\mathrm{est}}=R_{\mathrm{frame}}^{⊤}$.

### 2.3. Empirical Evaluation: Automatic Calibration

We evaluate the proposed Automatic Calibration framework through a comprehensive empirical study that reflects realistic operating conditions, including sensor noise, drift, and outliers. The experiments are designed to directly instantiate the problem formulation introduced in Section 2.2 and to demonstrate the practical effectiveness of using SO3_SPMC for automatic orientation calibration between heterogeneous IMU systems.

#### 2.3.1. Overview of the Automatic Calibration Method

Our experiment emulates how Automatic Calibration would be used in a real-world setting. In the experiment, participants performed a set of predefined activities (e.g., walking, typing, folding clothes) while wearing both DOT sensors (low cost IMU sensors needing calibration) and MVN sensors (measuring ground truth orientations). One or more of these activities can be *Calibration Tasks (CTs)*. The key assumption is that, for a given activity, the induced orientation distribution of a correctly calibrated sensor is a characteristic and repeatable structure on SO(3).

For a given DOT recording session, the orientation distribution generated during the CT serves as the source set B (Figure 2). A corresponding reference CT distribution obtained from the MVN system–either from prior sessions of the same participant or aggregated across multiple prior participants—serves as the target set A. Importantly, there is no one-to-one correspondence between elements of A and B.

This setup directly instantiates the problem described in Section 2.2, namely the alignment of two axis-consistent SO(3) distributions with unequal cardinalities. We employ the SO3_SPMC algorithm to estimate the rotation that best aligns B to A (Figure 2d). Once the alignment is obtained, the estimated rotation is applied to the entire DOT session, including both the CT and all other activities.

Evaluation is performed by comparing the calibrated DOT orientations against the corresponding MVN orientations. We report both the alignment accuracy with respect to the full MVN distribution and the calibration error relative to the session-specific MVN CT distribution, which serves as ground truth for calibration quality. While in our experiment we always collected both the ground truth (MVN) orientations and low-cost IMU (DOT) orientations, in a real-world deployment only the low-cost (DOT) sensors would be worn, and they would be calibrated via a reference CT distribution gathered ahead of time.

#### 2.3.2. Experiment Setup, Study Design, and Data Collection

To apply the proposed Automatic Calibration framework, we first construct a reference orientation distribution A corresponding to a chosen Calibration Task (CT). Data were collected using a set of 11 activities (see Appendix A), which participants performed during each recording session.

The dataset consists of recordings from five participants (P1–P5; two male and three female). Each participant completed the full activity sequence twice, resulting in two sessions per participant. We denote a session using the notation $P_{n}S_{m}$, where $P_{n}$ refers to participant *n* and $S_{m}$ refers to session *m*. All participants provided informed consent prior to data collection (Virginia Tech IRB #18-877).

Each recording session lasted approximately 20–25 min. Before each session, the experiment room was prepared with all necessary materials corresponding to the activity list. Participants wore a full XSens MVN Link motion capture suit, consisting of 17 IMUs. In addition, three XSens DOT sensors were securely attached using tape directly on top of the MVN sensors located on the pelvis, right forearm (RFA), and left forearm. This configuration ensured that MVN and DOT sensors experienced nearly identical physical motion while operating in independent reference frames. A detailed illustration of the sensor placement, activity setup, and room layout is shown in Figure 3.

#### 2.3.3. Evaluation Method

Following data collection, MVN and DOT data streams were synchronized. MVN data were recorded at 240 Hz and DOT data at 120 Hz. For analysis, both datasets were downsampled to 40 Hz and temporally aligned.

The synchronized data were manually segmented into subsets corresponding to the individual activities listed in Appendix A. For evaluation, we focus on data from the Pelvis and Right Forearm (RFA) sensors. For each activity and each sensor type, this process yields 20 datasets (five participants × two sessions × two systems).

For example, for Activity 1 and the Pelvis sensor, the resulting datasets include P1S1-MVN-Pelvis, P1S1-DOT-Pelvis, …, P5S2-MVN-Pelvis, P5S2-DOT-Pelvis. An analogous structure applies to the RFA sensor and the other activities. This preprocessing ensures that all datasets are temporally aligned, activity-consistent, and suitable for calibration and evaluation.

#### 2.3.4. Terminology and Definitions

We introduce the following definitions and terminology, which are used throughout the experimental analysis.

##### Normalized RFA (NormRFA) Distribution

The Normalized RFA (NormRFA) distribution represents the orientation of the right forearm relative to the pelvis. After synchronizing the Pelvis and RFA sensors, each frame contains paired orientations with identical timestamps. For each frame, the normalized RFA orientation is computed as

(3) $$R_{PB_{i}}={R^{-1}}_{GP}\cdot R_{GB_{i}},$$

where $R_{GP}$ denotes the pelvis orientation with respect to the global frame, and $R_{GB_{i}}$ denotes the *i*-th RFA orientation with respect to the global frame. This normalization procedure is consistent with prior work [42,45,46]. The resulting NormRFA distribution captures motion relative to the root segment and is invariant to global orientation.

##### Single Frame Calibration (SFC)

Single Frame Calibration (SFC) is a conventional calibration approach that aligns the DOT sensor frame to the MVN frame using a single synchronized reference frame, typically the initial frame of a particular activity. Given synchronized MVN and DOT orientations, the fixed calibration rotation is computed as

(4) $$\begin{array}{cc} R_{\mathrm{fixed}} & =R_{0}^{MVN}\cdot {\left(R_{0}^{DOT}\right)}^{-1}, \end{array}$$

(5) $$\begin{array}{cc} R_{i,\mathrm{SFC}}^{DOT} & =R_{i}^{DOT}\cdot R_{\mathrm{fixed}}. \end{array}$$

Here, $R_{0}^{MVN}$ and $R_{0}^{DOT}$ denote the initial orientations of the MVN and DOT sensors, respectively. While SFC ensures frame consistency at the reference frame, it does not account for time-varying sensor drift. The DOT sensors we used exhibited significant drift over time during some activities, especially when the wearer changed the orientation of their arms or pelvis frequently.

##### Mean Angular Error (MAE)

The Mean Angular Error (MAE) between two sets of orientations is computed as

(6) $$\mathrm{MAE}=\frac{1}{n}\sum_{i=1}^{n}\left|{\hat{q}}_{i}-q_{i}\right|,$$

where ${\hat{q}}_{i}$ and $q_{i}$ denote the quaternions from the two orientation sets being compared. The MAE is reported in degrees and is meaningful only when both sets are expressed in the same reference frame.

##### Definition of Sets A, B, $B_{\mathrm{Calib}}$, and $B_{\mathrm{GT}}$

The set A denotes the reference orientation distribution, here derived from MVN data. The set B denotes the uncalibrated DOT orientation distribution. After applying a calibration method (either SFC or SO3_SPMC), we obtain the calibrated set $B_{\mathrm{Calib}}$. The corresponding ground-truth distribution is denoted by $B_{\mathrm{GT}}$. In an ideal calibration scenario, the MAE between $B_{\mathrm{Calib}}$ and $B_{\mathrm{GT}}$ would be zero.

##### Sensor Drift and Calibration Error

When calibration is performed using SFC, the MAE between $B_{\mathrm{Calib}}$ and $B_{\mathrm{GT}}$ reflects accumulated sensor drift over time. In contrast, when calibration is performed using SO3_SPMC, the MAE quantifies the residual calibration error after automatic distribution alignment. Both quantities are evaluated using the same MAE metric but reflect different calibration mechanisms.

##### Orientation Signatures

Orientation Signatures are central to our Automatic Calibration framework. An Orientation Signature is defined as an aggregated SO(3) distribution constructed from MVN data collected across multiple sessions for a specific activity and sensor location (Pelvis, RFA, or NormRFA).

Before aggregation, all constituent datasets are transformed into a common reference frame. For example, to construct the *Pelvis Signature* for Activity 1, we aggregate MVN Pelvis data from all sessions (P1S1 through P5S2) after aligning them into a shared coordinate frame. The resulting signature represents an activity-specific orientation distribution that captures consistent motion-induced structure while averaging out subject- and session-specific noise.

Each Orientation Signature is visualized by converting the TBVs of its SO(3) distribution into 2D spherical histograms using the procedure described in Section 2.1.5. This yields three 2D distributions per activity, corresponding to the *x*-, *y*-, and *z*-axis TBVs. Using the same process, we construct *RFA Signatures* and *NormRFA Signatures* from their respective datasets.

Figure 4 shows an example of the Pelvis and NormRFA Signatures for Activity 2 (walking), and Appendix B shows the signatures for all of the activities. Comparing Orientation Signatures across activities provides insight into activity-dependent orientation patterns and enables the selection of suitable calibration tasks.

#### 2.3.5. Case Study Design

We design a series of case studies to systematically evaluate the behavior of standalone IMUs under drift and to assess the effectiveness of the proposed SO3_SPMC-based Automatic Calibration (AuC) framework. The goals of these case studies are threefold: (i) quantify sensor drift under conventional Single Frame Calibration (SFC), (ii) evaluate calibration performance under varying data availability conditions, and (iii) assess generalization across subjects and sessions.

For each activity, we define three sets: a reference set A, an uncalibrated set B, and a ground-truth set $B_{GT}$. Set B always consists of DOT data, while $B_{GT}$ consists of the corresponding MVN data. Set A may be chosen as MVN data from the same session, a different session, another subject, or an aggregated Orientation Signature, depending on the case. The alignment between A and B is performed using either SFC or SO3_SPMC (AuC), depending on the case. Below, we describe each case in detail.

**Case 1: Pelvis Sensor Drift | SFC.**

For each activity, we consider each MVN Pelvis dataset as A and the corresponding DOT Pelvis dataset as B. The ground-truth set $B_{GT}$ is identical to A. Applying SFC yields the Pelvis sensor drift (see “Sensor Drift and Calibration Error” in Section 2.3.4) for each activity. Error values are aggregated across all 10 sessions per activity.

**Case 2: Pelvis Sensor Calibration | Within-Subject and Within-Session AuC.**

This case uses the same datasets as Case 1, but replaces SFC with the AuC SO3_SPMC algorithm for alignment between A and B.

**Case 3: RFA Sensor Drift | SFC.**

This case mirrors Case 1 but uses RFA sensor data instead of Pelvis data.

**Case 4: RFA Sensor Calibration | Within-Subject and Within-Session AuC.**

This case mirrors Case 2 but uses RFA sensor data instead of Pelvis data.

**Case 5: Pelvis Sensor Calibration | Within-Subject and Between-Session AuC.**

Each participant performs all activities in two sessions. For each activity, MVN data from one session is selected as A, while DOT data from the other session is selected as B. The corresponding MVN dataset from the DOT session serves as $B_{GT}$. This case evaluates whether calibration generalizes across time for the same subject.

**Case 6: Pelvis Sensor Calibration | Pelvis Signature Excluding Subject Data AuC.**

This case evaluates cross-subject generalization. For each activity, A is defined as the Pelvis Signature with all data from the test subject removed. The test subject’s DOT data forms B, and the corresponding MVN data forms $B_{GT}$. This assesses calibration performance when no subject-specific data are available in the reference set.

**Case 7: RFA Sensor Calibration | Within-Subject and Between-Session AuC.**

This case parallels Case 5, using RFA sensor data instead of Pelvis data.

**Case 8: RFA Sensor Calibration | RFA Signature Excluding Subject Data AuC.**

This case parallels Case 6, using the RFA Signature instead of the Pelvis Signature.

**Case 9: NormRFA Calibration | SFC.**

This case mirrors Cases 1 and 3 but uses normalized RFA (NormRFA) distributions from MVN and DOT data, aligned using SFC.

**Case 10: NormRFA Calibration | Within-Subject and Between-Session AuC.**

This case mirrors Case 7, replacing raw RFA data with NormRFA data.

**Case 11: NormRFA Calibration | NormRFA Signature Excluding Subject Data AuC.**

This case mirrors Case 8, replacing raw RFA data with NormRFA data.

#### 2.3.6. Initial Mis-Calibration

Across ten recording sessions, after the MVN calibration procedure, the MVN Pelvis sensors exhibited mean orientation variability of 1.86°, 1.04°, and 1.75° along the *x*, *y*, and *z* axes, respectively. For the MVN RFA sensors, the corresponding values were 6.78°, 5.64°, and 5.16°. In contrast, the uncalibrated DOT Pelvis sensors showed substantially larger variability, with values of 70.12°, 67.31°, and 19.04°, while the DOT RFA sensors exhibited variability of 27.16°, 27.12°, and 18.84° along the respective axes. These results highlight the need for a robust automatic calibration mechanism for standalone IMUs.

## 3. Results

### 3.1. Results of the Case Studies

#### 3.1.1. Calibration Error Computation

For each case study and each activity, we compute the calibration error using the procedure outlined in Algorithm 2. Depending on the case, alignment between the reference set A and the source set B is performed using either Single Frame Calibration (SFC) or the proposed SO3_SPMC-based Automatic Calibration (AuC). The resulting calibrated set $B_{Calib}$ is then compared against the ground-truth set $B_{GT}$ using Mean Angular Error (MAE).
**Algorithm 2** Case Study Analysis1:**procedure** ComputeCalibError($A,B,B_{GT}$)2:      **if** SFC **then**3:            $B_{Calib}\leftarrow$ SFC(A,B)4:      **else**5:            $B_{Calib}\leftarrow$ SO3_SPMC(A,B)6:      **end if**7:      Error← MAE$\left(B_{Calib},B_{GT}\right)$8:      **return** Error9:**end procedure**

For each activity and case, we aggregate the calibration errors across all relevant sessions and visualize the distributions using box plots. The results for all activities and cases are summarized in Figure 5.

#### 3.1.2. Comparison of SFC and AuC (Cases 1–4)

Cases 1 and 2 (Pelvis) and Cases 3 and 4 (RFA) directly compare Single Frame Calibration (Cases 1, 3) against the proposed SO3_SPMC-based Automatic Calibration (Cases 2, 4) using identical input data. Across nearly all activities, AuC yields lower median calibration errors and substantially tighter error distributions.

For the Pelvis sensor, Case 2 consistently outperforms Case 1. Median errors in Case 2 (AuC) range from 0.18° (Activity 1) to 5.54° (Activity 7), whereas Case 1 (SFC) exhibits larger errors, ranging from 0.34° to 16.77°. Activity 7 involves significant upper-body motion, which induces drift in the Pelvis sensor under SFC. AuC effectively compensates for this drift, reducing the error by nearly a factor of three.

A similar trend is observed for the RFA sensor. Case 4 (AuC) achieves errors as low as 0.16° (Activity 1) and up to 15.31° (Activity 8), while Case 3 (SFC) reaches errors as high as 35.44°. Activity 8 (preparing breakfast) contains highly variable hand motions, which leads to severe drift of the DOT sensors over time; this shows up as large errors with the SFC. Despite the ongoing sensor drift, AuC substantially reduces the calibration error, demonstrating robustness to motion-induced orientation variability.

#### 3.1.3. Within-Subject, Between-Session Calibration (Cases 5 and 7, AuC)

Cases 5 and 7 evaluate whether Automatic Calibration generalizes across different recording sessions of the same participant. Both cases achieve their lowest errors for Activity 1 (N-Pose), as expected due to minimal motion. Case 5 (Pelvis) achieves a median error of 0.8°, while Case 7 (RFA) achieves 1.65°.

For the Pelvis sensor (Case 5), calibration errors remain below 10° for several dynamic activities, including Activities 3, 4, 5, 6, and 8. Notably, Activities 4 and 5 yield median errors of 5.31° and 5.02°, respectively. In contrast, Case 7 shows reduced robustness for the RFA sensor, failing to achieve sub-10° median error for any activity other than Activity 1. Typing and mouse-use activities (Activities 3 and 4) perform best out of these cases, with median errors in the range of 15–16°.

#### 3.1.4. Cross-Subject Calibration Using Orientation Signatures (Cases 6 and 8, AuC)

Cases 6 and 8 assess the ability to calibrate a participant’s DOT data using Orientation Signatures that explicitly exclude that participant’s data. This setting evaluates the generalization capability of the proposed approach.

For both Pelvis (Case 6) and RFA (Case 8), Activity 1 again yields the lowest calibration errors, with medians of 0.67° and 1.04°, respectively. Case 6 maintains median errors below 10° for Activities 2, 3, 4, 5, and 8, indicating strong cross-subject generalization for the Pelvis sensor. However, Case 8 struggles to achieve comparable performance for the RFA sensor, with only Activity 3 (typing) yielding relatively moderate error ($17.2^{{}^{\circ}}$). This highlights the increased variability of distal limb motion and its impact on cross-subject calibration.

#### 3.1.5. Normalized RFA Calibration (Cases 9–11)

Cases 9–11 examine calibration performance using normalized RFA (NormRFA) distributions. Case 9 applies SFC, while Cases 10 and 11 employ SO3_SPMC (AuC) for between-session and signature-based calibration, respectively.

Interestingly, Cases 10 and 11 exhibit highly similar performance, suggesting that NormRFA-based calibration generalizes well across both sessions and subjects. For both cases, calibration errors remain below 10° for Activities 1 and 4. Activity 1 yields the lowest errors, at 1.10° (Case 10) and 1.11° (Case 11), while Activity 4 achieves errors of approximately 9.5°. Other activities, including walking, typing, and reading, show median errors in the range of 14–16°. Comparable trends are observed in Case 9, indicating that normalization mitigates some—but not all—effects of motion-induced drift.

### 3.2. Automatic Calibration Applied Across Full Sessions

#### 3.2.1. Overview

Based on the analysis in Section 3.1, we select Activities 2, 3, and 4 as candidate Calibration Tasks (CTs), and include Activity 1 (N-Pose) as a baseline for comparison. For each session, Automatic Calibration (AuC) is executed once using data from the selected CT, and the resulting calibration is applied to the entire session. Performance is then compared against Single Frame Calibration (SFC), which is performed using the first frame of the session and held fixed throughout.

For Activity 1, calibration is performed at the beginning of the session, following the standard protocol illustrated in Figure 2. For Activities 2, 3, and 4, calibration occurs mid-session, during execution of the selected CT. This setup allows us to assess the effectiveness of AuC when calibration is performed after drift has already accumulated.

#### 3.2.2. Method

Calibration performance must be interpreted in the context of time-dependent sensor drift. Since AuC is executed partway through the session, its performance reflects both prior drift (before calibration) and subsequent drift (after calibration). To provide a fair comparison, we evaluate AuC relative to SFC applied at the start of the session, which reflects common practice in IMU-based data collection.

For each selected activity, we evaluate calibration for the Pelvis, RFA, and NormRFA representations. Sets A and B are constructed following the methodology of Cases 6, 8, and 11 for Pelvis, RFA, and NormRFA data, respectively. After estimating the calibration rotation, the entire DOT session is transformed into the calibrated reference frame, as illustrated in Figure 2d. In parallel, SFC is applied once at the beginning of the session and held constant.

This procedure is applied to all ten data sessions. For each session and activity, we compute the difference between the MAE obtained using SFC and that obtained using AuC. This comparison essentially removes the effects of sensor drift, since the drift occurs equally for both SFC and AuC. Thus, the numbers directly indicate how accurate AuC is relative to SFC. Detailed per-session drift statistics for the Pelvis and RFA sensors are reported in Table A1 in Appendix C.

#### 3.2.3. Results with Full Sessions

Figure 6 summarizes the difference in calibration error between AuC and SFC across activities and sensor representations. Positive values indicate that AuC outperforms SFC, while negative values indicate the opposite.

For Activity 1 (N-Pose), AuC performs comparably to SFC. This outcome is expected, as sensor drift is minimal at the beginning of the session. In several instances, AuC slightly outperforms SFC, consistent with the within-session calibration results observed in Cases 1–4 (Figure 5).

For Activity 2, AuC yields lower median error than SFC for the RFA and NormRFA representations, with median improvements of 0.9° and 0.7°, respectively. In contrast, SFC performs slightly better for the Pelvis sensor, with a median difference of −1.8°. Although the median differences are small, the error distributions exhibit substantial variability, ranging approximately from −20° to +10°, indicating that either method may outperform the other depending on session-specific drift characteristics.

For Activities 3 and 4, AuC exhibits slightly higher median error than SFC across Pelvis, RFA, and NormRFA representations, with median differences ranging from −1.1° to −5.3°. However, error distributions remain wide, particularly for the RFA and NormRFA representations, where relative differences exceed ±20° in some sessions. These results suggest that while mid-session calibration can mitigate accumulated drift, its effectiveness depends on both the selected calibration task and the motion characteristics of the sensor location.

Although activity labels are provided in the present study, the proposed framework is not restricted to manually segmented activities. The same Orientation Signature representation used for calibration could also be used to identify candidate calibration tasks in unlabeled streams by comparing sliding-window signatures against a library of activity-specific reference signatures. In this sense, activity recognition and calibration can be coupled: candidate windows are first identified from the IMU stream, and SO3_SPMC is then applied only when the window matches a known activity signature with sufficient confidence.

In the present evaluation, we intentionally instantiate AuC using a single calibration event to provide a controlled comparison against SFC. This evaluation choice does not restrict the proposed framework to one-shot calibration. In deployment, AuC can operate as a routine and repeated recalibration procedure over sliding or event-triggered windows. Each accepted window would produce a local calibration estimate, and subsequent accepted windows could update the calibration as additional drift or slippage occurs.

Overall, these results demonstrate that Automatic Calibration can achieve performance comparable to, and in some cases exceeding, Single Frame Calibration when applied to full-session data. At the same time, they highlight the inherent trade-offs associated with mid-session calibration in the presence of time-varying drift and complex motion patterns.

## 4. Discussion

Based on the experimental results, we draw several key observations regarding sensor drift, calibration accuracy, and the conditions under which Automatic Calibration is most effective.

*Observation 1: Motion-induced drift is sensor- and activity-dependent.* Standalone DOT sensors exhibit significantly larger drift during activities involving frequent, rapid, or random motion. This effect is particularly pronounced for distal sensors such as the Right Forearm (RFA). As observed in Cases 1 and 3, RFA drift is highest during activities with substantial arm motion (e.g., Activities 7, 8, and 9), and substantially lower during activities with limited arm movement (e.g., Activities 1, 3, and 4). Across all activities, Pelvis sensors consistently exhibit lower drift than RFA sensors, which is expected given the reduced motion of the pelvis relative to the upper limbs.

*Observation 2: SO3_SPMC effectively mitigates drift when correspondence exists.* When a one-to-one correspondence exists between MVN and DOT data, SO3_SPMC consistently reduces calibration error relative to Single Frame Calibration (SFC). This is evident in Cases 2 and 4, where SO3_SPMC outperforms SFC across most activities. Unlike SFC, which relies on a single initial frame and therefore accumulates error as drift increases over time, SO3_SPMC leverages the distribution of orientations over time. By aligning average motion-induced structure rather than individual frames, SO3_SPMC reduces the effect of drift on the calibrated result.

*Observation 3: Within-subject data yields better calibration accuracy than cross-subject signatures for raw sensors.* For unnormalized sensor data, calibration using between-session data from the same subject yields lower error than calibration using subject-excluded signatures. This trend is consistently observed when comparing Case 5 with Case 6 (Pelvis) and Case 7 with Case 8 (RFA). The improved performance is attributed to subject-specific motion characteristics, which are preserved across sessions but diluted in cross-subject signature distributions.

*Observation 4: Normalized representations improve calibration robustness.* Calibration based on normalized RFA (NormRFA) distributions consistently outperforms calibration using raw RFA data. Cases 10 and 11 exhibit lower errors across most activities compared to Cases 7 and 8. Normalization removes the effect of global body orientation changes, as both Pelvis and RFA sensors rotate together. This suggests that normalized representations are better suited for calibration in wearable sensing scenarios, where relative motion with respect to the body frame is often of primary interest.

*Observation 5: Activity structure strongly influences calibration quality.* Activities characterized by minimal motion or highly synchronized movement are the most suitable for Automatic Calibration. Across all case studies, Activity 1 (N-Pose) consistently yields the lowest calibration errors, effectively replicating fixed-pose calibration. However, because Activity 1 is equivalent to an explicit calibration posture, its practical advantage is limited.

For individual sensor calibration, Activities 4, 3, 5, and 2 (Computer Mouse Movement, Typing, Reading, and Walking, respectively) emerge as the next most reliable Calibration Tasks. In these activities, Pelvis calibration errors remain below 10°, while RFA errors typically fall in the 15–$25^{{}^{\circ}}$ range. These errors are largely attributable to drift accumulated during the activity itself, affecting both SFC and Automatic Calibration. Activities 6, 7, 8, and 9 are the least suitable for calibration due to highly variable and unsynchronized motion.

When using normalized RFA data, the ranking of effective activities improves, with Activities 4, 3, 2, and 5 achieving errors below 16°. In this setting, Activity 8 remains the least suitable calibration candidate. Given that wearable sensing applications typically operate in a body-centric reference frame, normalized representations are likely the most practically relevant.

### 4.1. Full-Session Calibration Performance

Results from full-session calibration experiments (Section 3.2, Figure 6) show that Automatic Calibration achieves performance comparable to SFC. In some cases, such as Activity 2 using NormRFA, Automatic Calibration yields lower median error (by 0.73°), while Activities 3 and 4 show similar performance between the two methods. Across all activities, error differences vary widely, reflecting the highly session-dependent nature of sensor drift. In extreme cases, RFA sensors drifted by 60–90° over a single session (Table A1, Appendix C). These results suggest that Automatic Calibration is competitive with SFC even under severe drift, and may benefit further from sensors with improved long-term stability.

### 4.2. Limitations and Future Directions

The proposed framework has several limitations that motivate concrete methodological extensions. First, the current implementation assumes that calibration windows correspond to known activities. In deployment, Automatic Calibration should operate as a routine and repeated recalibration procedure: the sensor stream would be continuously monitored for motion segments that provide reliable calibration information. Candidate windows could be proposed by lightweight IMU-based activity detectors for common actions such as walking, typing, or mouse use, and then verified using the SO(3) orientation signatures introduced in this work. In this formulation, activity recognition and calibration are coupled: the activity detector identifies candidate windows, while signature alignment determines whether the window is sufficiently informative for calibration. The SPMC correlation peak, the sharpness or entropy of the TBV distributions, and the consistency among the independently aligned *x*-, *y*-, and *z*-axis distributions provide natural confidence measures for accepting or rejecting a calibration window.

Second, although the present evaluation estimates a single calibration rotation from one selected activity segment for controlled comparison with Single Frame Calibration, the method is not inherently one-shot. A practical long-duration system would use a sliding-window or event-triggered strategy in which every reliable activity signature produces a candidate calibration rotation. Repeated walking, typing, or mouse-use segments would therefore yield a time-indexed sequence of calibration estimates on SO(3). These estimates could be fused using a Lie-group filter or smoother, with the calibration state modeled as a slowly varying rotation and each accepted SO3_SPMC alignment treated as a noisy observation. Confidence-weighted updates would allow reliable windows to correct drift that accumulates after previous calibration events, while suppressing ambiguous or poorly conditioned segments that may lead to inaccurate sensor calibrations. In offline processing, accepted calibration events could also be geodesically interpolated or smoothed over time, avoiding the need to apply a single fixed calibration to the entire recording. Additionally, in rehabilitation applications and other cases where a person’s motion patterns are likely to change over time, it will be necessary to update the reference activity signatures gradually to reflect the changing motion patterns.

Finally, our results indicate that calibration reliability depends on sensor location and motion structure. Pelvis and normalized body-centric representations are more stable, whereas distal limb sensors exhibit larger cross-subject and activity-dependent variability. Future work should therefore investigate multi-sensor and kinematically constrained calibration, where stable body segments provide reference structure for more variable sensors such as the forearm or wrist. We note that the current study only included five individuals; larger-scale studies with more participants, sensor placements, attachment conditions, and longer recordings are also needed to quantify the generalization of learned orientation signatures. Additionally, future studies are needed to evaluate the generalizability of this method to more diverse populations, including individuals with different ages, gait patterns, motor abilities, and clinical conditions. Together, these directions move the proposed distribution-alignment framework toward a confidence-aware, repeatedly invoked calibration module for long-term wearable sensing.

## 5. Conclusions

We presented an Automatic Calibration framework for aligning the orientations of standalone, low-cost IMU sensors using activity-specific orientation distributions, without requiring explicit calibration poses or one-to-one correspondence between measurements. By formulating calibration as the alignment of SO(3) distributions induced by known activities, our approach leverages the repeatable structure of human motion rather than individual sensor frames.

The proposed method employs SO3_SPMC to robustly align orientation distributions in the presence of noise, outliers, and sensor drift. Through extensive experimental evaluation involving multiple subjects, activities, and calibration scenarios, we demonstrated that Automatic Calibration achieves performance comparable to conventional Single Frame Calibration. It is particularly useful when drift has accumulated or when normalized representations are used.

Our results show that activities characterized by minimal or synchronized motion yield the most reliable calibration, while distal limb sensors are more sensitive to motion-induced drift. We further showed that normalized, body-centric representations significantly improve calibration robustness and generalization across sessions and subjects. Importantly, the proposed framework enables calibration using mid-session activities, providing flexibility beyond traditional start-of-session calibration protocols.

Although this work focuses on body-worn IMUs, the underlying formulation is sensor-agnostic and is applicable to any system that produces orientation measurements in SO(3). As such, the proposed approach has potential applications in wearable sensing, robotics, and human–machine interaction, where low-cost sensors and long-duration operation are common.

Future work will explore automatic identification of calibration activities, integration of multiple calibration events over time, and extension of the framework to additional sensing modalities.

## Figures and Tables

**Figure 1:**
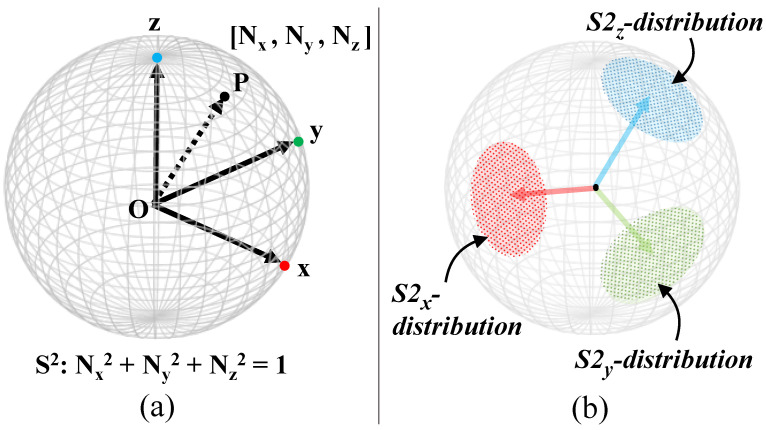
$S^{2}$ representation of a normal vector and $S^{2}$ representation of the sets of Transformed Basis Vectors (TBVs). (**a**) In $S^{2}$ representation, we view an arbitrary normal vector $\vec{OP}$ as a point *P* on the surface of the unit sphere; *P* is the S2-point of $\vec{OP}$. Black arrows indicate the reference-frame directions (equivalently, the TBVs of the identity rotation). The red (*x*), green (*y*), and blue (*z*) dots are the S2-points of the identity rotation. (**b**) The $S2_{x}$, $S2_{y}$, $S2_{z}$ distributions are colored red, green, and blue, respectively; their mean directions are shown as arrows.

**Figure 2:**
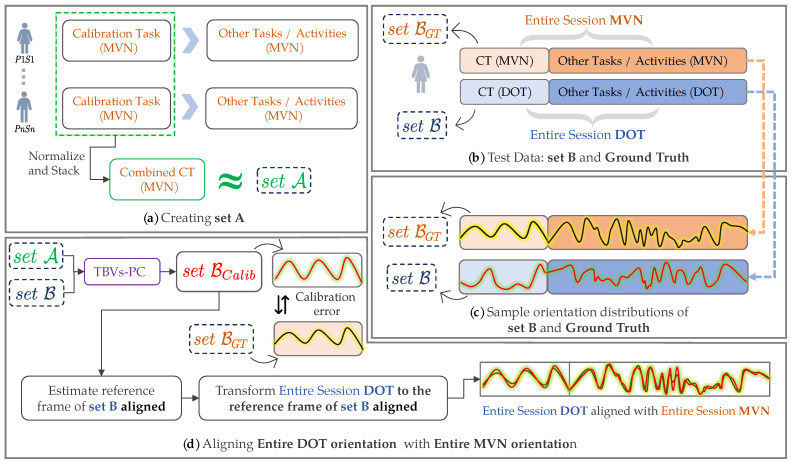
Overview of the Automatic Calibration framework. (**a**) A reference orientation distribution A is constructed from correctly calibrated MVN data during a particular Calibration Task, potentially aggregated across subjects and sessions. (**b**) A test scenario in which a participant wears both MVN and DOT sensors. The MVN data is used as the Ground Truth (GT), while the DOT sensor data is uncalibrated. (**c**) Orientation distributions induced by the test scenario. The wavy lines indicate an example sensor orientation over time. Note that the orientations from the DOT sensors do not match the Ground Truth since they are not calibrated. (**d**) The reference distribution A and the observed DOT distribution B are aligned to estimate a calibration rotation; when distribution B is rotated to form distribution $B_{\mathrm{Calib}}$, this will have a small error relative to the Ground Truth $B_{\mathrm{GT}}$ that depends on how well the distributions were aligned and how similar the reference distribution A matches the orientations in the Ground Truth distribution $B_{\mathrm{GT}}$. The calibration rotation is then applied to the entire DOT session.

**Figure 3:**
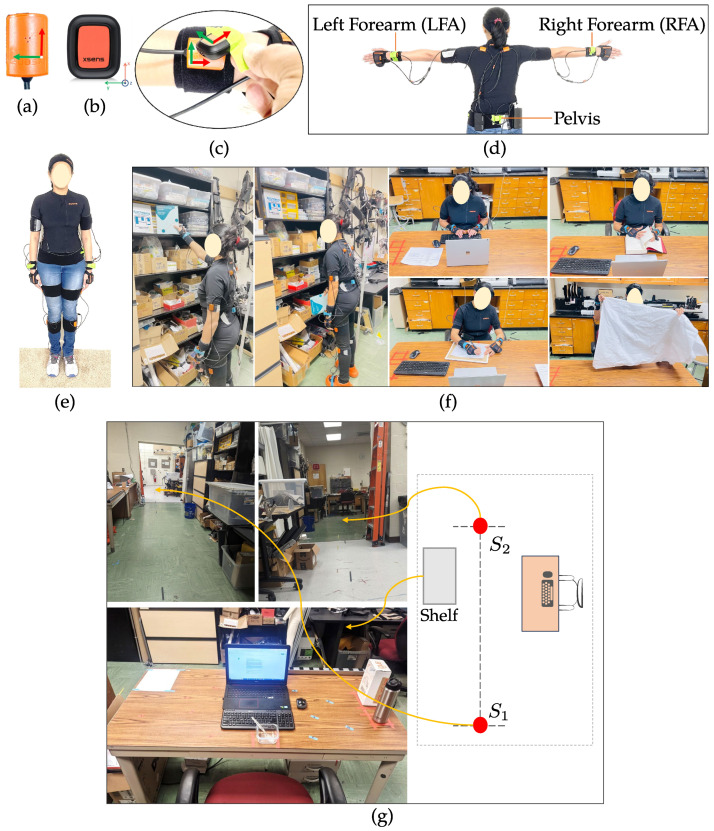
Experimental setup and study design. In (**a**,**b**), we show the local coordinate system of the MVN and DOT sensors, respectively. In (**c**,**d**) we show detail on the locations of the DOT sensors. In (**e**), a participant is shown standing in the N-Pose, which represents Activity 1 from the activity list (Appendix A). In (**f**), the same participant is engaged in various other activities, including organizing books, typing, writing, reading, folding clothes, and more. In (**g**), we present a layout of the experiment room on the right side, showcasing the arrangement of the two stations, $S_{1}$ and $S_{2}$. The lab environment and the setup of the tables are also shown.

**Figure 4:**
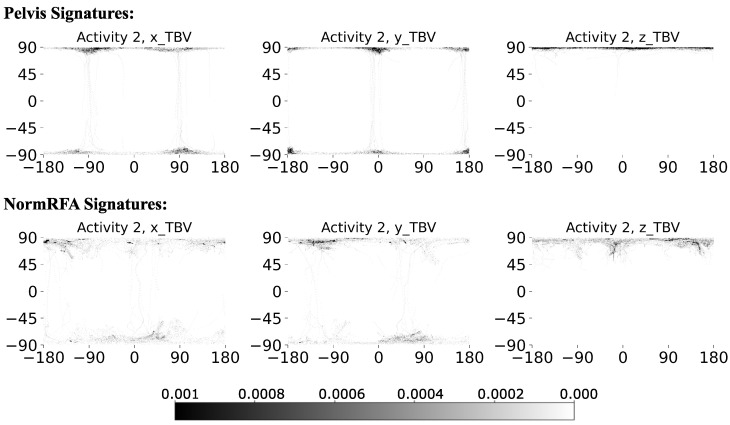
Pelvis and NormRFA Signatures for Activity 2 (walking). Each of the signatures shows a cluster of points near the top and bottom of the graph. We note that in the SO3_SPMC algorithm, the centroid of the points is mapped to be at the north pole; here, since there is some spread in the points, the cluster shows up at both +90 and −90 degrees, and is spread fairly evenly across the different longitudes (*x*-axis values). There are denser clusters of points at some longitudes (e.g., around −90 and +90 degrees for *x*-TBV), which correspond to more common angles in the periodic motion of the body during walking. The NormRFA signatures show much more variation than the Pelvis since the pelvis moves through a fairly small angular range while walking, while the arms swing back and forth.

**Figure 5:**
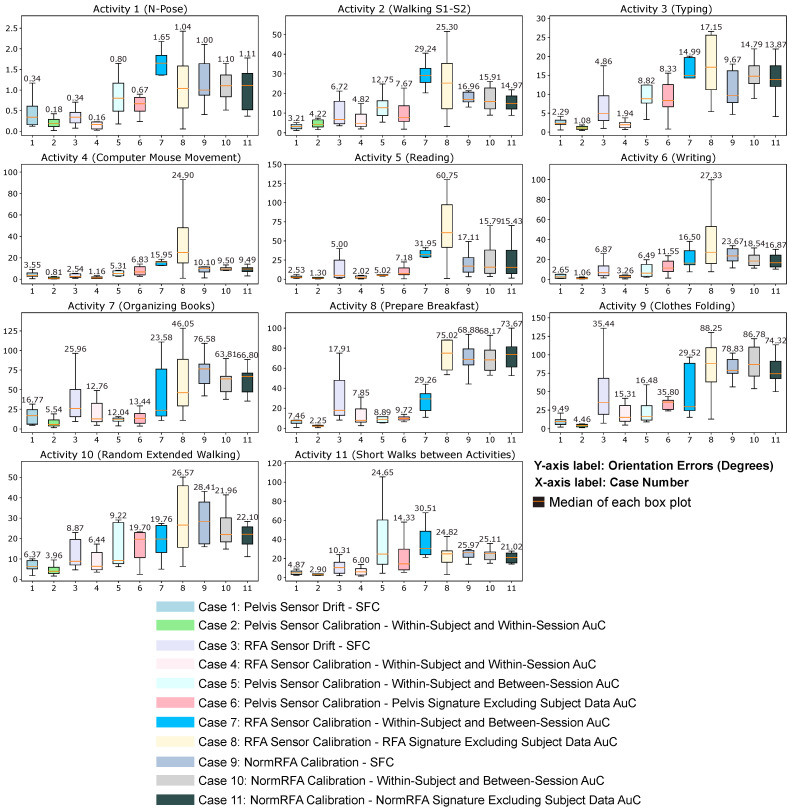
Comparison of calibration error across activities and case studies. Each subplot corresponds to one of the 11 activities, where the data is only from within that activity. The *x*-axis denotes the Case number (see Section 2.3.5), and the *y*-axis shows orientation error in degrees.

**Figure 6:**
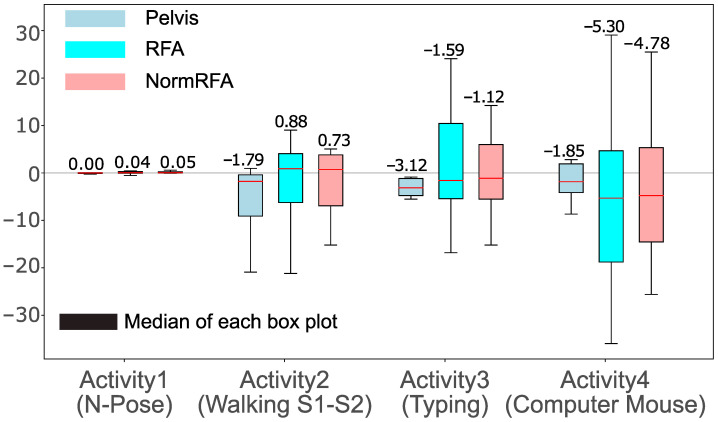
Difference in calibration error between Automatic Calibration (AuC) and Single Frame Calibration (SFC) when applied to full data collection sessions. Positive values indicate lower error with AuC, while negative values indicate lower error with SFC. For each activity, the reference set A is constructed as an activity-specific signature excluding the test subject. The estimated calibration is applied to the entire DOT session and compared against SFC applied at the beginning of the session.

**Figure A1:**
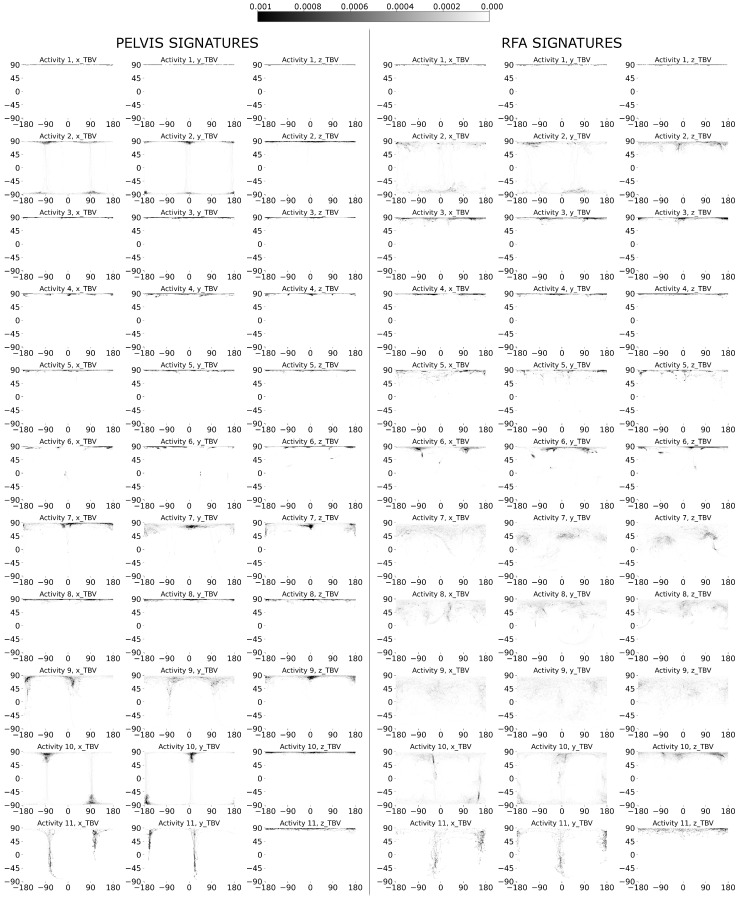
The orientation signatures for both the Pelvis and NormRFA are shown for each activity, as outlined in “Orientation Signatures” in Section 2.3.4. For each activity, we have a signature SO(3) distribution. From this, we extract the TBVs, and the orientation signatures are then visualized as 2D histograms of these three TBVs. The x-axis of these histograms represents the range of longitude angles from −180° to +180°. Meanwhile, the y-axis corresponds to latitude angles, ranging from −90° to +90°. These histograms are constructed with a bin size of (180, 360) along the y and x axes, respectively.

## Data Availability

The raw data supporting the conclusions of this article will be made available by the authors on request.

## Appendix A. Activity List

At the beginning of each session, participants were provided with the instructions for the task. Throughout the session, participants rarely adjusted the positions of the sensors. There were few periods of motionlessness (rest time) in the sequence where participants were either standing or sitting in a natural pose while listening to instructions. The following is a list of description of activities included in the study. ET stands for Estimated Time, which is the approximate time to complete the activity.

- **Activity 1 (Staying in N-Pose):** *Instruction:* Stay in N-Pose for 20-30 sec at station $S_{1}$ facing towards $S_{2}$.
- **Activity 2 (Walking Back and forth):** *Instruction:* Walk in a natural pose back and forth. Start from $S_{1}$, proceed to $S_{2}$, turn around, and return back to $S_{1}$. *Observation:* Different participants exhibited varied walking patterns, particularly in their hand movements, during the walking activity. Notably, different hand movements were observed even during the two sessions performed by a single participant. ET: 45 s.
- **Activity 3: Typing** *Instruction:* Type a paragraph 3 times in a text processor. *Observation:* Participants exhibited hand movements while typing. Different participants displayed distinct typing patterns. For instance, some preferred typing with the keyboard close to the edge of the table, while others chose to type with a slight elevation. ET: 3 min.
- **Activity 4: Mouse Movement** *Instruction:* Draw three smiley faces in the Microsoft Paint app. Play the Minesweeper game online at the preferred difficulty level for three minutes. *Observation:* Participants displayed occasional random hand movements during the activity. ET: 2 min.
- **Activity 5: Reading** *Instruction:* Pick a book of your choice from the table, then read it for 5 min. *Observation:* Participants exhibited different hand motions while reading. For example, some individuals turned over the pages frequently, while others assumed different hand poses. Notably, different hand movements were observed even during the two sessions performed by a single participant. ET: 2 min.
- **Activity 6: Writing** *Instruction:* Pick a notebook and a pen from the table. Then, write the given paragraph three times. *Observation:* Participants exhibited different hand motions while writing. Some individuals preferred keeping their hand straight relative to their chest, while others preferred writing at an angle. ET: 3 min.
- **Activity 7: Organizing Books** *Instruction:* From station $S_{2}$, proceed to the nearby shelf. Pick a book from a lower section of the shelf and place it on an upper shelf. Participants are allowed to use both hands if required. Repeat this process at a natural interval for three minutes. *Observation:* Participants exhibited different hand motions while organizing. Additionally, some participants had to bend more than others, and some even had to squat in order to pick up the books. ET: 3 min.
- **Activity 8: Prepare and Eat Breakfast** *Instruction:* From station $S_{2}$, proceed to the table. Take a seat and adjust it. Take a milk bottle and pour cereal into a bowl. Mix the cereal and milk, then eat. *Observation:* Participants exhibited different hand motions while organizing and performing the activity. It is worth noting that even during the two sessions conducted by a single participant, distinct hand movements were observed. ET: 3 min.
- **Activity 9: Folding and Unfolding Clothes** *Instruction:* Stand up from the chair. Take the unfolded apron from the table. Fold the apron neatly and place it in a designated corner of the table. Repeat this process three times. *Observation:* Participants exhibited mostly random hand motions while performing this activity. ET: 3 min.
- **Activity 10: Extended Walking** *Instruction:* After completing activity 9, proceed to station $S_{2}$. Walk randomly within the room. There are no restrictions on hand motion. *Observation:* Participants exhibited different hand motions while walking during this activity. ET: 1.5 min.
- **Activity 11: Short Walk Between Activities** This is not a listed activity. However, we extracted frames where participants moved between stations or different locations such as the table or shelf.

## Appendix B. Data Signatures

Images showing the orientation signatures from the Pelvis and Right Forearm are shown in Figure A1.

## Appendix C. Sensor Drift Amounts

We present the data of the average error for how much the Pelvis and RFA sensors drifted over the course of the entire session in Table A1. This gives some insight into the magnitude of the sensor drift, in comparison to the benefits of using the Automatic Calibration versus Single Frame Calibration. In the Table, the data is the error over the whole session, but using Activity 1 (staying in N-Pose) for the calibration.

**Table A1:** Calibration Errors (deg) for the Automatic Calibration (AuC) and Single Frame Calibration (SFC) methods for the entire sessions, using Activity 1 as the calibration task.

	Pelvis		RFA	
Session	AuC	SFC	AuC	SFC
P1S1	11.8	11.8	20.7	20.8
P1S2	2.9	2.9	27.7	28.1
P2S1	21.4	23.3	7.5	6.9
P2S2	10.3	10.3	17.9	17.9
P3S1	10.3	10.3	28.8	29.1
P3S2	9.8	9.8	19.7	20.0
P4S1	7.3	7.4	58.8	58.7
P4S2	36.0	35.7	76.6	76.3
P5S1	7.9	7.9	81.8	82.0
P5S2	13.6	13.5	92.3	92.3
